# Cytotoxic and Anticancer Effects of ICD-85 (Venom Derived Peptides) in Human Breast Adenocarcinoma and Normal Human Dermal Fibroblasts

**Published:** 2019

**Authors:** Peyman Kheirandish Zarandi, Abbas Zare Mirakabadi, Fattah Sotoodehnejadnematalahi

**Affiliations:** a *Department of Biology, Science and Research Branch, Islamic Azad University, Tehran, Iran.*; b *Department of Venomous Animal and Antivenom Production, Razi Vaccine and Serum Research Institute, Agriculture Research Education and Extension Organization (AREEO), Karaj, Iran.*

**Keywords:** ICD-85, MCF-7, HDF, Caspase-9, MTT assay

## Abstract

ICD-85 (venom derived peptides) has anti-proliferative effect and anti- angiogenesis activity on cancer cells. This study was performed to test the effect of ICD-85, on Human breast adenocarcinoma (MCF-7) and normal Human Dermal Fibroblasts (HDF) cell lines. In this experimental study, Mitochondrial activity, Neutral red uptake, Lactate dehydrogenase (cell necrosis), and cell morphology were assessed under unexposed and ICD-85 exposed conditions. Caspase-9 colorimetric assay kit was used to determine caspase protease activity. Morphological changes in MCF-7 cells on treatment with ICD-85 compared with untreated MCF-7 cells are consistent with characterizing the features of apoptosis such as granulation and cell rounding which finally results in the generation of apoptotic bodies. In contrast, this difference was not observed in normal cells. In MTT assay, ICD-85 induced dose dependent manner cytotoxic effects on MCF-7 cells which were confirmed by neutral red assay. The results showed that inhibitory concentration 50% (IC50) value of ICD-85 for MCF-7 cells at 24 h was 36.45 ± 0.38 μg/mL. However, when HDF cells were exposed to ICD-85, no significant elevation of LDH release were observed at concentrations below 20 μg/mL. The apoptosis-induction of ICD-85 on MCF-7 cell was found to be through activation of caspase-9 which was 13 fold greater than unexposed cell. This study showed that ICD-85 induced apoptosis in MCF-7 cell line through caspase activation and hence it can be considered for further investigation to use ICD-85 as a potential therapy for breast cancer.

## Introduction

Nowadays, cancer is one of the major public health problems (1). Breast malignant neoplastic disease is one of the most frequent malignancies in women from developed and undeveloped states (2, 3). Despite huge efforts to find a cure for breast cancer, current treatments such as chemotherapy, radiation therapy, immunotherapy, gene therapy, endocrine therapy, and combination of surgery (breast-conserving surgery or mastectomy) have still achieved satisfactory levels of protection or remission (4-6).

The undesirable side effects of chemotherapy can be nonselective distribution of drugs, multidrug resistance, increased drug toxicity, adverse side effects to normal tissues and non-response noted the inherent usefulness of cytotoxic anti- cancer drug (7). Target treatments were aimed to block specific biologic transduction pathways or cancer proteins. The molecular targets include receptors, growth factors, apoptosis, and angiogenesis that are associated kinase cascade or molecules (8).

Nowadays, oncologists look for new anticancer drugs with fewer side effects (9). Venom of scorpions and snakes are part of biological resources as well as the future prospects for the treatment of some incurable diseases. The venoms are complex mixture of many different toxins, low molecular weight peptides, and different enzymes with high molecular activities. The activities have been alternated for the development of anticancer agents (10, 11). Some of venoms had been reported which have anticancer activity through induction of apoptosis in target cells (12, 13).

Polypeptides derived from scorpion venom in East Asia have antiproliferative and apoptotic effect on human prostate cancer cells (DU-145) (14). Apoptogenic peptides isolated from *Tityus discrepans *scorpion venom have anticancer effects against the breast cancer cell line SKBR3 and no effect on the normal monkey kidney cell line (MA104). The results showed that Apoptogenic peptides bind to SKBR3 cell surface and induce FasL and BcL-2 expression (15). The existence of a cytotoxin protein NN-32 has been reported in the venom of Indian spectacled cobra (*Naja naja*) that induces apoptosis on human leukemic U937 cells (16).

Zare Mirakabadi *et al. *reported the cytotoxicity of ICD-85 (venoms derived peptides) on MDA-MB-231 (a highly invasive breast cancer) and HeLa (cervical adenocarcinoma) cell lines. It is believed that the cytotoxic action of the enzyme in this component is mainly modulated by apoptosis (17, 9). On the other hand, previous *in-vitro *studies had shown the ICD-85, as an anticancer agent, anti- proliferative effect, and anti-angiogenesis activity on cancer cells through the induction of apoptosis without significant effect on normal cells (18, 19). In another study, ICD-85 was able to suppress the breast tumor in mice (20). The aim of the present study is to further investigate cytotoxicity and mode of cell death caused by ICD-85 against human breast adenocarcinoma (MCF-7) and to compare it with normal Human Dermal Fibroblasts (HDF) cell lines using various techniques.

## Experimental

*Chemicals*

RPMI-1640 medium, Dulbecco′s modified Eagle′s medium (DMEM) (Gibco, USA), Penicillin/streptomycin solutions, Fetal bovine serum, Trypsin-EDTA (Invitrogen, USA), MTT 3-(4,5-dimethyl-thiazol-2-yl)-2,5-diphenyltetrazolium bromide, Neutral red dye (NR) Sigma (St. Louis, MO, USA), Dimethyl sulfoxide (DMSO) (Sigma-Aldrich, USA), Cytotoxicity Detection Kit of lactate dehydrogenase enzyme (LDH) Pars Azmoon (Tehran, Iran), Caspase-9 colorimetric assay Kit BioVision (USA) were of analytical grade and purchased locally.

**Figure 1:**
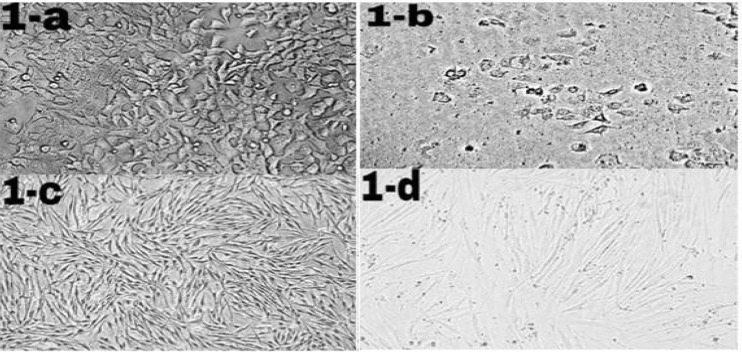
Morphological changes of ICD-85 on MCF-7 and HDF cells. MCF-7 and HDF cells (1 × 104 cells/well) were cultured in RPMI-1640 and DMEM medium respectively supplemented with 10% heat inactivated fetal bovine serum and treated in the absence (control cells) or presence of ICD-85 at 80 μg/mL for 24 h at 37 °C. Morphological changes of treated cells were observed with an invert microscope and compared with control cells. Figure 1b (20X) shows granulation and cell rounding in MCF-7 cells treated with ICD-85 as compared to untreated MCF-7 cells (1-a, 20X). There are no significant morphological changes in HDF normal cells treated with ICD- 85 (1-d, 20X) as compared to untreated HDF cells (1-c, 20X)

**Figure 2:**
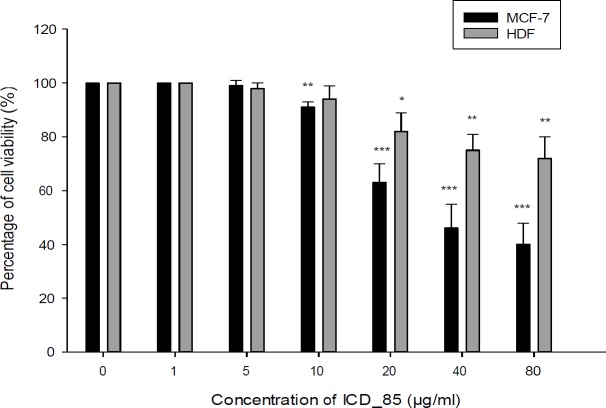
Cell viability of the MCF-7 and HDF cells after treatment with ICD-85. The cytotoxicity was evaluated by the MTT method 24 h after treatment of the cell lines with ICD-85 (1–80 μg/mL). The results were shown as mean ± SD of three independent experiments (n = 3). Doses of 10 µg/mL or greater, significantly killed MCF-7 cell compared to the control ^**^P < 0.01

**Figure 3:**
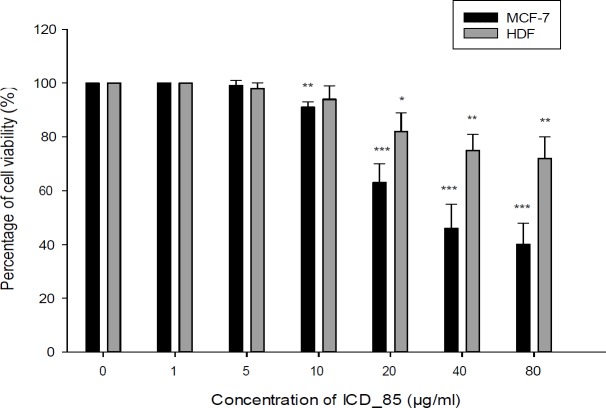
The neutral red uptake assay to assess the cell viability of MCF7 and HDF cells incubated with various concentrations of ICD-85 (1–80 μg/mL) for 24 h. The data were presented as mean ± SD (n = 3). Doses of 5 µg/mL or greater, significantly killed MCF-7 cell compared to the control ^*^P < 0.05

**Figure 4:**
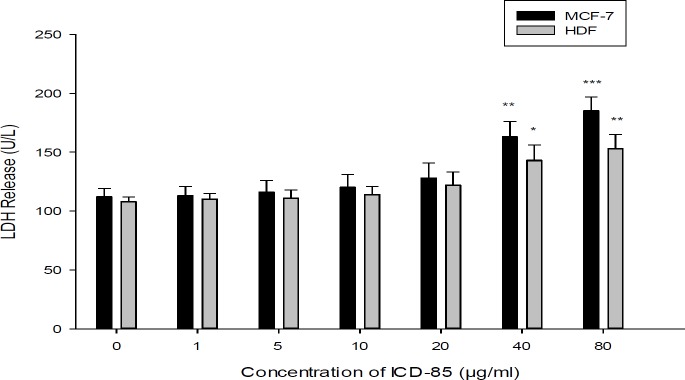
Effect of ICD-85 on LDH leakage in MCF-7 and HDF cells. The cells were treated with different concentrations of ICD-85 for 24 h. At the end of the incubation period, the LDH assay was performed to assess the LDH leakage as described in methods section. The data were expressed as the mean ± SD of three independent experiments carried out in triplicate. Significances were indicated in comparison to control.^*^P < 0.05, **P < 0.01, ^***^P < 0.001

**Figure 5:**
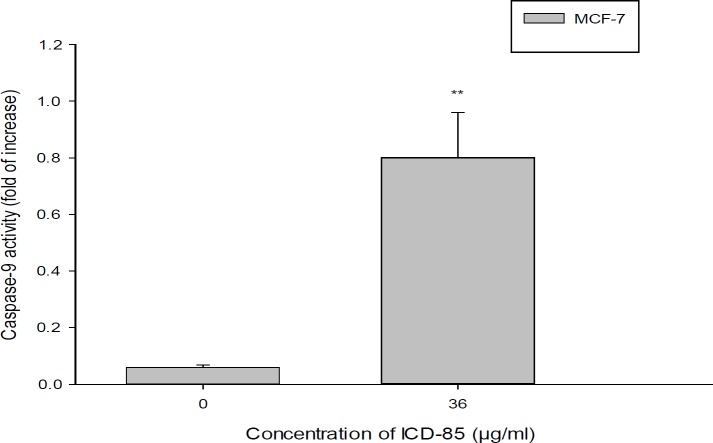
Determination of caspase-9 activity in MCF-7cell treated with ICD-85. For evaluation of caspase-9 activity, MCF-7 cell were treated in the absence and in the presence of IC50 concentration (36.45 ± 0.38 μg/mL) of ICD-85 for 24 h. The datashown are the means ± SD of three independent experiments. Significances were indicated in comparison to control. **P < 0.01

*ICD-85 (venom-derived peptides)*

The active fraction of ICD-85 were combinated of three peptides, ranging in size from 10000 to 30000 Da and being derived of the venoms from Iranian brown snake (*Gloydius halys*) and yellow scorpion (*Hemiscorpius lepturus*). The peptides were selected based on a study of crude venom cytotoxicity and isolation of cytotoxic peptides by two-step purification that included gel filtration and HPLC. The lyophilized peptides was stored at −20 °C and solubilized in phosphate buffered saline (PBS) immediately before its use in the tests.

*Cell culture*

Human breast adenocarcinoma (MCF-7) and normal Human Dermal Fibroblasts (HDF) cell lines were purchased from Pasteur Institute of Iran and cultured according to the manufacturer’s instruction. The cells were propagated in 25 mL plastic flasks in RPMI-1640 and DMEM medium respectively. The Media were supplemented with 10% heat inactivated fetal bovine serum (FBS) along with 100 U/mL penicillin and 100 µg/mL streptomycin. The culture was incubated at 37 °C with 95% humidity air that contained 5% CO2.

*Mitochondrial activity*

Mitochondrial function, as an indicator of cytotoxicity, was assessed by measuring the capacity of MCF-7 and HDF cells to reduce MTT to formazan (21). Briefly, MCF-7 and HDF cells were seeded in 96-well plates at a density of 1 × 104 cells per well and grew to 75% confluence. The cells were treated with different concentrations (1, 5, 10, 20, 40 and 80 µg/mL) of ICD-85 and incubated for 24 h. After treatment, 20 μL of MTT stock solution (5 mg/mL) was added to each well and the cells were incubated for 3 h at 37 °C. The solution was then discarded and 100 μL of Dimethyl sulfoxide was added into each well and incubated for 15 min in the dark to dissolve insoluble formazan crystals. Immediately, the absorbance of each well was read at 570 nm using an ELISA reader. 

The percentage of the viable cells was calculated according to the following formula: Percentage of viability of each concentration = (corrected mean OD of test/corrected mean OD of control) × 100.

*Neutral red uptake assay (NRU assay)*

Neutral red uptake assay was done to determine the accumulation of neutral red dye in the lysosomes of viable, uninjured cells (22). MCF-7 and HDF cells were seeded in 96-well plates at a density of 1 × 104 cells/well. The cells were treated with various concentrations (1, 5, 10, 20, 40 and 80 µg/mL) of ICD-85 and incubated for 24 h. After overnight, the wells medium was replaced with a new one containing NR (40 μg/mL). After 3 h of incubation, neutral red medium was removed and the cells were washed with PBS for the remaining dye. Finally, neutral red destain solution (50% from ethanol 96%, deionized water 49% and glacial acetic acid 1%) was added to each well and the plate was gently shaken for 20 min. Optical density (OD) of neutral red extract was read with Synergy HT Microplate Reader (Bio-Tek Instruments, Winooki, VT) at 540 nm.

*Morphologic Analysis Using an Inverted Microscope*

Morphological studies using a normal inverted microscope were carried out to observe the morphological changes of cell death in cancer and normal cells treated with ICD-85. The untreated cells were served as the negative control.

*Measurement of cell necrosis (Lactate dehydrogenase assay)*

LDH is a cytoplasmic enzyme retained by viable cells with intact plasma membranes, but released from necrotic cells with damaged membranes. HDF, MCF7 cells were plated at a density of 1 × 104 cells per well in 96-well cell culture plates, and incubated for 24 h. Post media changes, and treatments of the cells at ICD-85 concentrations (1, 5, 10, 20, 40, and 80 µg/mL) that the cells were incubated at 37 °C for 24 h. After incubation, the released LDH in the media was measured with the Cytotoxicity Detection Kit (Pars Azmoon, Tehran, 

Iran).

*Caspas-9 Assay*

The Caspase-9 activity was determined by Caspase Colorimetric Assay Kit (according to the manufacturer’s instructions, BioVision, USA). Therefore, MCF-7 cells (3 × 106) were harvested and lysed in 5 mL of chilled lysis buffer and then incubated on ice for 10 min. The suspension was centrifuged for 1 min at (10,000 × g) and the supernatant (cytosolic extract) was transferred to a fresh tube. The protein concentration assay was then performed by Lowry assay. 200 μg cytosolic protein was diluted to 50 μL cell lysis buffer for each assay in a 96- well plate. Each reaction buffer which containing 10mM dithiothreitol (DTT) and 5 μL of the 4 mM LEHD-pNA substrate was added into each well of the designated enzyme assay and the plate was then incubated at 37 °C for 2 h. At the end of incubation, the plate was read in a microtiter plate reader at 405 nm. The caspase activity increase was determined by comparing the results of treated samples with the level of the uninduced control.

*Statistical Analysis*

The values are expressed as means ± SD of three repeats in each group. The data were analyzed using student′s *t*-test with statistical significance for *P *< 0.05.

## Results

*Morphologic Analysis Using an Inverted Microscope*

MCF-7 and HDF cells were exposed to ICD-85 for 24 h and morphological changes were examined using invert microscopy. The morphology of control (unexposed to ICD-85) MCF-7 and HDF cells were shown in (Figure 1a) and (Figure 1c) respectively. While significant morphological changes in MCF-7 cancer cells were observed after ICD-85 treatment, characterizing the features of apoptosis such as granulation and cell rounding (Figure 1b), no significant morphological alterations were found in HDF cells treated with same concentration of ICD-85 (Figure 1d).

*Mitochondrial activity*

The cytotoxic effects of various concentrations of ICD-85 on MCF-7 and HDF cells were measured by the MTT assay during 24 h exposure. The results showed decreased MCF-7 cells viability after the treatment with ICD-85 in a dose dependent manner. The viability of MCF-7 cells after exposure with 1, 5, 10, 20, 40, and 80 μg/mL of ICD-85 was 100, 99, 91, 63, 46, and 40% respectively whereas cell viability of HDF after treatment with 1, 5, 10, 20, 40, and 80 μg/mL of ICD-85 was, 100, 98, 94, 82, 75, and 72% respectively (Figure 2). The IC50 value for MCF-7 was estimated to be 36.45 ± 0.38 μg/mL. ICD-85 also inhibited the growth of HDF cells. However, the concentration inhibition of ICD-85 on HDF cells was comparatively lower than MCF-7 cells (Figure 2).

*Neutral red uptake assay (NRU assay)*

As shown in Figure 3, the percentage of viable cells of MCF-7 after exposure with 1, 5, 10, 20, 40 and 80 μg/mL of ICD-85 decreased to 100, 95, 87, 54, 33, and 28%, respectively. In contrast with cell survival of HDF after treatment with 1, 5, 10, 20, 40, and 80 μg/mL of ICD-85 was 100, 97, 93, 73, 63, and 60% respectively. The IC50 value of ICD-85 for MCF-7 cells at 24 h was estimated and found to be 21.97 ± 0.63 μg/mL. On the other hand, ICD-85 showed negligible growth inhibitory effect on HDF cells at concentrations less than 20 μg/mL as compared to unexposed cells (Figure 3).

*Measurement of cell necrosis (Lactate dehydrogenase assay)*

Although treatment of MCF-7 cells with ICD-85 at concentrations of 1, 5, 10, and 20 μg/mL did not significantly increase LDH release, but when ICD-85 concentration increased to 40 μg/mL and above, the LDH activity in the culture media increased significantly (*P *< 0.01 and *P *< 0.001) respectively (Figure 4).

*Caspas-9 Assay*

The activation of caspase-9 in MCF-7 cells exposed to ICD-85 was investigated. As shown in Figure 5, the caspase activity of cells exposed to 36 µg of ICD-85 was elevated by about 13.

## Discussion

Some of scorpion and snake venoms have the property of inhibiting the growth of various types of cancers. Several antitumor peptides have been reported from scorpion and snake venoms showing cytotoxic activities (23, 24). This study was designed to examine the cytotoxic and anti-cancer properties of ICD-85 (venom- derived peptides) in human breast adenocarcinoma (MCF-7) cells. The active peptides in ICD-85 were isolated from the Iranian snake (*Gloydius halys*) and scorpion (*Hemiscorpius lepturus*) (17).

Results of our study showed that ICD-85 induced of morphological alterations such as cell shrinkage and cell rounding in MCF-7 cells. In this study, most of the MCF-7 cells exposed with ICD-85 showed cell shrinkage, which can be important marker of apoptosis. It seems that morphological changes are consistent with an apoptotic mechanism of cell death. In contrast, no morphological changes observed in normal HDF cells that were exposed to ICD- 85 at concentration similar to MCF-7 cells. These results are supported by our previous studies on MDA-MB231 cell line exposed to ICD-85 which showed the shrinkage of the cells under light microscopic (17).

The results from the mitochondrial activity assays indicated that ICD-85 is able to decrease the survival of MCF-7 cells in a dose-dependent manner. The treatment of MCF-7 cells with 80 μg/mL of ICD-85 for 24 h reduced the survival rate of cells to 40%, whereas, in contrast, the survival of HDF cell at same concentration of ICD-85 was above 72%. The lower cytotoxicity in human non- tumourigenic cell line (HDF) when compared to tumourigenic (MCF-7) cell line, suggests a selective cytotoxic property of the ICD-85 against tumourigenic cell. The results of MTT on the MCF-7 cells were confirmed by neutral red uptake assay.

The LDH activity of culture MCF-7 cells exposed to ICD-85 were determined and no significant effects were observed in the LDH release at concentrations below of 20 μg/mL. The LDH leakage assay is due to cell membrane damage. It is clear that LDH levels (as a marker of necrosis) in the cell medium elevated after the cells were exposed to the anticancer agents (25). The increase in LDH activity of MCF-7 cells exposed to high concentrations of ICD-85 may be due to necrotic effect of ICD-85 at high concentration. On the other hand, the HDF cells exposure with ICD-85 at concentration lower than 40 μg/mL showed no significant rise in LDH levels.

Previous studies by Zare Mirakabadi *et al. *showed that the ICD-85 has no effect on normal cells (MRC5), at low concentrations (16 and 26 μg/mL) (19). The results of caspase 9 activity of cells exposed to ICD-85 in comparison to non- exposed cells showed a 13 fold increase in the activity of caspase. Caspase-9 as an indicator of apoptosis may play a critical role in determination of apoptotic nature of a drug. Numerous toxins and proteins have been reported to induce apoptosis in the cancer cell determined by caspase-9 (26-28).

The results of this study showed that the ICD-85 induced apoptosis to MCF-7 cell lines through caspase activation and hence it could be considered for further investigation on consideration of ICD-85 as a potential therapy for breast cancer.
